# Factors associated with adverse outcome among children with sickle cell disease admitted to the pediatric intensive care unit: an observational cohort

**DOI:** 10.1186/s13613-024-01283-5

**Published:** 2024-04-10

**Authors:** Michaël Levy, Jérôme Naudin, Guillaume Geslain, Arielle Maroni, Bérengère Koehl, Fleur Le Bourgeois, Géraldine Poncelet, Maryline Chomton, Anna Deho, Sébastien Julliand, Stéphane Dauger, Julie Sommet

**Affiliations:** 1 Pediatric Intensive Care Unit, Assistance Publique-Hôpitaux de Paris, Robert-Debré University Hospital, Université Paris Cité, Paris, France; 2 Reference Center for Sickle-Cell Disease, Robert-Debré University Hospital, Assistance Publique-Hôpitaux de Paris, Inserm U1134, Université Paris Cité, Paris, France; 3 Pediatric Mobile Emergency Unit, Robert-Debré University Hospital, Assistance Publique- Hôpitaux de Paris, Université Paris Cité, Paris, France; 4 General Pediatrics, Pediatric infectious disease and Internal Medicine, Robert-Debré University Hospital, Assistance Publique-Hôpitaux de Paris, ECEVE UMR 1123, Paris, France

**Keywords:** Anemia, Acute chest syndrome, Non-invasive ventilation, Respiration, Artificial, Pediatrics, Prognosis, Mortality, Blood transfusion

## Abstract

**Background:**

Sickle cell disease (SCD) is one of the most frequent inherited diseases in the world. Over the last decades, in high-income countries, an important decrease in mortality have been observed due to the improvement of care. However, children with SCD can become critically ill and require admission in Pediatric Intensive Care Units (PICU). The purpose of this study was to describe the epidemiology of children with SCD admitted to PICU for acute crisis and to identify factors associated with adverse outcome (AO).

**Methods:**

We conducted a retrospective study in a Tertiary Hospital in France including all consecutive children with SCD admitted to PICU between January 1st, 2009 and December 31, 2019. We collected baseline patient’s characteristics, clinical and biological data as well as treatments and life sustaining therapies used in the PICU. Patients were defined as experiencing AO in case of death during stay and/or need for invasive mechanical ventilation (MV) and/or for non-invasive ventilation (NIV) for more than 3 days and/or need for vasopressors and/or need for renal replacement therapy.

**Results:**

We included 579 admissions in 395 patients, mainly of SS genotype (90%) with a median age of 9.2 years [5.5–13.4] and a median baseline hemoglobin of 8.0 g/dl (7.5–8.8). The two main reasons for admission were acute chest syndrome (ACS) (*n* = 331, 57%) and vaso-occlusive crisis refractory to first line therapy (*n* = 99, 17%). Half of patients required NIV and 47 (8%) required MV. The overall length of stay was 3 days [1–4] and seven (1%) patients died during PICU stay.There was a total of 113 (20%) admissions with AO and on multivariable analysis, baseline hemoglobin < 8 g/dL, history of bronchial obstruction and admission for ACS were associated with AO. There was no difference in the proportion of hydroxyurea treatment or exchange transfusion program between patients with AO and the other patients.

**Conclusions:**

Baseline hemoglobin < 8 g/dL, history of bronchial obstruction and admission for ACS were the strongest risk factors for severe evolution in SCD children admitted to PICU. These factors could be taken into consideration when choosing the adequate therapeutic options.

**Supplementary Information:**

The online version contains supplementary material available at 10.1186/s13613-024-01283-5.

## Introduction

Sickle cell disease (SCD) is one of the most common genetic diseases in the world and the number of people living with sickle cell disease increased by more than 40% in 2021 compared with 2001 [1, 2]. Over the past few decades, newborn screening along with prophylactic penicillin, parental education, anti-pneumococcal vaccination, hydroxyurea, advances in transfusion safety, and hematopoietic stem cell transplantation (HSCT), have led to a decrease in mortality in high-income countries [1, 3, 4]. However, children with SCD experience significant morbidity and healthcare utilization. Children with SCD may become critically ill due to acute crisis (AC) and require admission in the pediatric intensive care unit (PICU). In adults, only a few articles have studied SCD patients admitted to the ICU. Factors associated with adverse outcomes were high respiratory rate, low mean arterial blood pressure, acute kidney injury and red blood cell exchange transfusion before admission whereas results were controversial regarding hemoglobin levels [5, 6] Large cohorts of hospitalized children with SCD have been published [7] but only a few studies have described specifically SCD patients admitted to the PICU. Ettinger et al. in a large multicenter retrospective study found that patients requiring invasive mechanical ventilation (MV) had higher odds ratio for mortality, but this study did not provide a detailed description of the PICU stay [8]. The other studies to date included very few patients [9–11] and robust data on the epidemiology of SCD patients admitted to PICU and risk factors for adverse outcomes are lacking.

As the probability of being transferred to PICU at least once during the two first decades of life with SCD is high, these data appear crucial to help identifying the most severe patients. In fact, these patients could benefit from alternative therapy like early non-invasive ventilation (NIV) [12] or even tocilizumab (ClinicalTrials.gov Identifier: NCT05640271) to prevent adverse transfusion outcome and decrease the risk of post transfusion alloimmunization. Thus, the main objective of this study was to describe the epidemiology of children with SCD in the PICU and to define factors associated with adverse outcome (AO).

## Methods

### Study design and population

We conducted a single center retrospective study in Robert-Debré University Hospital PICU, Paris, France. This hospital has the largest cohort of pediatric SCD patients in France with more than 2000 patients. The study was approved by the Committee for the Evaluation of the Ethics of Research Projects of the Robert-Debré Hospital, Paris (n° 2022 − 635). We included all PICU admissions of patients aged from 0 to 20 years with SCD between January 1, 2009, and December 31, 2019. Patients with sickle cell trait or those admitted after HSCT were not included.

Patients were identified in the hospital database by selecting the International Classification of Diseases,10th Revision codes D57.0 (Hb-SS disease with crisis), D57.21 (Sickle-cell/Hb-C disease with crisis), D57.43 (Sickle-cell thalassemia beta zero with crisis), D57.45 (Sickle-cell thalassemia beta plus with crisis) and D57.81 (Other sickle-cell disorders with crisis).

### Data collection

The following data were collected from the medical records for each patient: gender, age, sickle-cell genotype, baseline hemoglobin level, selected medical history (including G6PD deficiency, history of acute chest syndrome (ACS), cerebral vasculopathy, stroke, bronchial obstruction, hydroxyurea treatment, exchange transfusion), unit of origin, completion of blood transfusion before PICU admission to treat the ongoing acute episode, reason for PICU admission (ACS, vaso-occlusive painful crisis (VOPC) refractory to standard therapy, stroke, splenic sequestration (SS), sepsis, perioperative, severe acute anemia not related to SS and other [1]), mortality and organ dysfunction scores (PIM-2 and PELOD-2-H24). Given the retrospective design of the study, compliance with treatments could not be assessed. Baseline hemoglobin level refers to hemoglobin level during SCD follow up quoted into the medical chart at least 3 weeks apart from any clinical event or any treatment intensification and at least 3 months after transfusion [13]. Bronchial obstruction refers to any bronchial obstruction in medical history quoted in medical chart and was defined according to age: 3 separate episodes of wheezing in children younger than 3 years, wheezing or coughing with bronchial obstruction confirmed by age specific tests in children between 3 and 6 years of age [14] and children older than 6 years [15].

During PICU stay, biologic data and treatments including life-sustaining interventions were collected: lowest hemoglobin (g/dL), highest white blood cell count (/mm^3^), highest CRP dosage (mg/L), hyponatremia < 135 mmol/L, acute kidney injury according to the KDIGO classification, blood support (transfusion, exchange transfusion), antibiotics, NIV, MV, fluid resuscitation and vasoactive drugs. Finally, PICU length of stay (PICU-LOS), vital status at PICU discharge and at 6 months after hospital discharge were also reported.

Patients were classified as having AO if they required NIV > 3 days [16], MV (excluding the first 24 h after surgery), vasopressors, renal replacement therapy or if they died in the PICU.

In our institution, NIV was indicated in case of ACS with signs of respiratory distress and/or increasing oxygen requirements or in case of any other respiratory distress due to other diseases causing hypoxemia or hypercapnia. In a previously published multicenter study on the use of NIV in ACS patients in 2 PICUs, most patients required less than 3 days of NIV [16]. For VOPC standard of care in pediatric ward included multimodal analgesia (acetaminophen, nefopam, morphine), hydration and transfusion in case of acute anemia at the discretion of the physician. In case of VOPC refractory to the precited therapy, patients were admitted to the PICU for ketamine treatment. Regarding exchange transfusions, indications were stroke, severe ACS, VOPC refractory to all antalgic therapies, thrombotic events, and surgery.

### Statistical analyses

The results of the descriptive analysis are expressed as numbers and percentages for qualitative variables, and as medians with quartiles [q1 and q3] and (minimum, maximum) for quantitative variables. Variables were compared between the two groups (patients with AO and others) by using the chi-square test or Fisher’s exact test for categorical variables and non-parametric test for continuous variables (Mann-Whitney-Wilcoxon).

To identify variables potentially associated with AO, univariate analyses were first performed. Variables with clinical relevance and a 20% level univariate analyses were selected and entered into a multivariable logistic model for repeated data. We used the Genmod procedure in SAS with a repeated statement to account for correlation of individuals in cases of multiple admissions. These were, G6PD deficiency, baseline hemoglobin < 8 g/dL, medical history of bronchial obstruction and admission diagnosis. A backward selection process was performed.

Statistical analysis was performed with SAS 9.3 software (Cary, NC, USA). All tests were bilateral, and p-values of 0.05 or less denotes statistical significance. The relative risks are expressed as odd ratio (OR) with 95% confidence interval (_95%_CI).

## Results

**Fig. 1:**
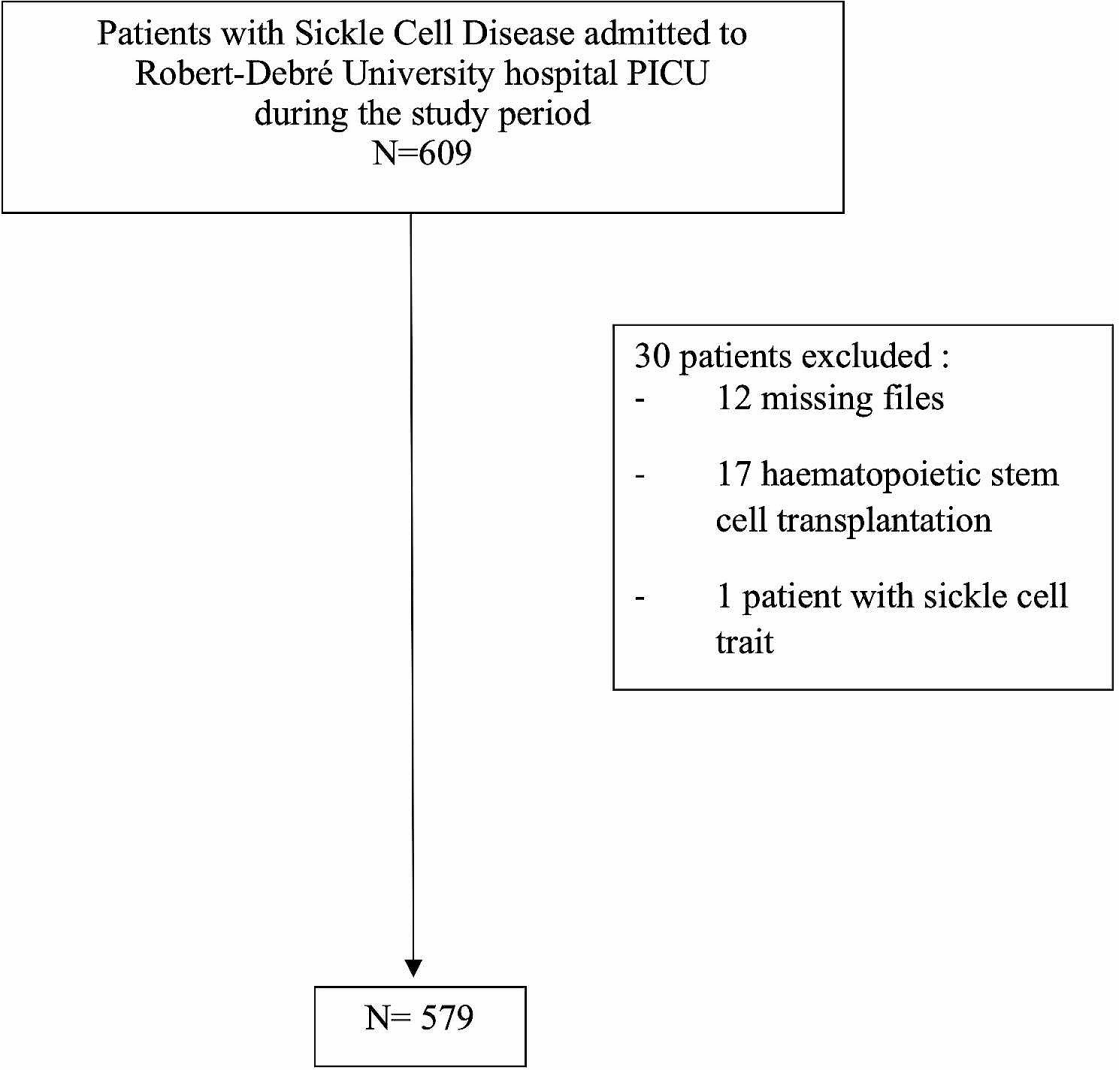
Flow chart

During the study period, 609 admissions of patients with SCD were included. After exclusion of 30 admissions, mainly because of admission following HSCT (Fig. 1), 579 stays of SCD patients were included with a median of 52 patients admitted per year [43.5–62.0]. There was a total of 395 distinct patients including 281 patients (71%) admitted only once during the study period and 114 patients (29%) admitted more than once in the PICU. The median age was 9.2 years [5.5–13.4] (0.2–19.2) and 60% of patients (*n* = 349) were males. The sickle cell genotype was SS in 93% of cases (*n* = 536), with a median baseline hemoglobin of 8.0 g/dl [7.5–8.8] (5.0-11.5). 51% (*n* = 283) were treated with hydroxyurea and 15% (*n* = 84) had associated G6PD deficiency (Table 1). Most patients were transferred from the general pediatric wards (*n* = 437, 75%) mainly for ACS (*n* = 331, 57%) and VOPC (*n* = 99, 17%). Half of patients required NIV with a median duration of 2 days [2–3] (0–9). The PICU-LOS was 3 days [1–4] (0–57).

**Table 1:** Baseline characteristics of patients with sickle cell disease admitted to the Pediatric Intensive Care Unit

	All patients (*N* = 579)	Patients with adverse outcome (*N* = 113)	Patients without adverse outcome (*N* = 466)	MD	p-value
Baseline characteristics					
Genotype, n (%)					
SS	536 (93%)	108 (95%)	428 (92%)	0	NS
SC / Sβ°	23 (4%) / 20 (3%)	4 (4%) / 1 (1%)	19 (4%) / 19 (4%)	0	
Age (years)	9.2 [5.5–13.4] (0.2–19.2)	10.1 [5.9–13.7] (0.3–19.7)	9.1 [5.3–13.3] (0.2–19)	0	NS
Male gender, n (%)	349 (60%)	73 (65%)	276 (60%)	0	NS
G6PD deficiency, n (%)	84 (15%)	22 (20%)	62 (14%)	13	NS
Baseline hemoglobin					
SS/Sβ°	8.0 [7.5–8.8] (5.0-11.5)	8.0 [7.5–8.5] (5.5–10.0)	8.2 [7.8-9.0] (5.0-11.5)	30	< 0.01
SC	10.5 [10.0–11.0] (8.0-12.5)	11.0 [10.0-10.5] (8.0-11.5)	10.5 [10.0–11.0] (8.0-11.5)	3	NS
Baseline hemoglobin < 8 g/dl	278 (51%)	66 (63%)	212 (48%)	33	< 0.01
History of bronchial obstruction, n (%)	137 (25%)	37 (35%)	100 (23%)	37	0.01
History of acute chest syndrome, n (%)	289 (51%)	56 (51%)	233 (51%)	12	NS
History of cerebral vasculopathy, n (%)	80 (16%)	12 (13%)	68 (17%)	74	NS
History of stroke, n (%)	43 (8%)	3 (3%)	40 (9%)	20	*†*
Hydroxyurea treatment, n (%)	283 (51%)	54 (52%)	229 (51%)	24	NS
Exchange transfusion program, n (%)	107 (19%)	19 (18%)	88 (19%)	17	NS
History of PICU admission	242 (47%)	48 (49%)	194 (47%)	64	NS
Unit of origin, n (%)				1	NS
General pediatrics department	437 (75%)	86 (76%)	351 (75%)		
Others	142 (25%)	27 (24%)	115 (25%)		
Emergency department	114 (20%)	17 (15%)	97(22%)		
Operating room	12 (2%)	5 (4%)	7 (2%)		
Home	6 (1%)	0 (0%)	6 (1%)		
Long term facility	3 (1%)	0 (0%)	3 (1%)		
Other PICU	6 (1%)	5 (1%)	1 (0%)		
**Diagnosis on admission, n (%)**				0	< 0.01
Acute chest syndrome	331 (57%)	80 (71%)	251 (54%)		
Other causes	248 (43%)	33 (29%)	215 (46%)		
Vaso-occlusive pain crisis	99 (17%)	2 (2%)	97 (21%)		
Stroke	39 (7%)	7 (6%)	32 (6%)		
Sepsis	18 (3%)	10 (9%)	8 (2%)		
Splenic sequestration	12 (2%)	2 (2%)	10 (2%)		
Peri-operative	10 (2%)	4 (4%)	6 (1%)		
Acute anemia*	9 (2%)	0 (0%)	9 (2%)		
Other	61 (11%)	8 (7%)	53 (11%)		
**PIM 2**	1.2 [0.8–3.2] (0-22.1)	3.3 [1.7–4.7] (0-22.1)	1.0 [0.8–2.8] (0-18.2)	59	< 0.01
**PELOD 2 (day 1)**	0 [0–1] (0–45)	1 [0–3] (0–33)	0 [0–0] (0–45)	59	< 0.01

PICU : pediatric intensive care unit ; MD : Missing Data ; NIV : non-invasive ventilation ; MV : mechanical ventilation; NS : non-significant ; PIM 2 : Pediatric Index of Mortality II ; PELOD2: Pediatric Logistic Organ Dysfunction 2. Adverse outcome (AO) was defined in case of NIV > 3 days (11), IMV (excluding the first 24 h after surgery), vasopressors, renal replacement therapy or in case of death in the PICU. *Acute anemia refers to an Hb level decrease ≥ 20% versus the baseline value and due to any reasons excepted splenic sequestration (Delayed Hemolytic Transfusion Reaction, Parvovirus infection…) † Irrelevant statistical testing due to reduced effectives. Quantitative data are reported as median [interquartile range] (extreme values)

**Fig. 2:**
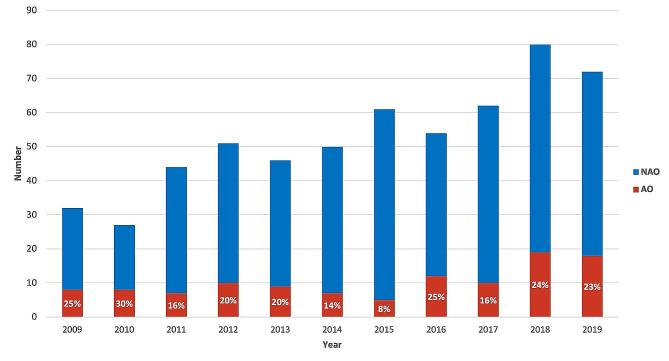
Proportion of patient admitted to the Pediatric Intensive Care Unit developing adverse outcome AO: adverse outcome, defined if the patient died during ICU stay and/or required invasive mechanical ventilation and/or non-invasive ventilation for more than 3 days and/or vasopressor and/or renal replacement therapy. NAO: non adverse outcome

There was a total of 113 (20%) patients with AO including 89 (79%) patients requiring NIV for more than 3 days, 47 (8%) requiring MV and 12 (2%) treated by vasoactive drugs. Seven patients with AO (6%) died in the PICU: three sepsis (one invasive pneumococcal disease and two severe malaria), one refractory acute respiratory distress syndrome complicating ACS, two massive strokes and one pulmonary embolism. The annual proportion of AO was similar during the study period (Fig. 2).

On univariate analysis, patients with AO had more frequently a baseline hemoglobin < 8 g/dl, a medical history of bronchial obstruction, more frequent admission for ACS and higher predicted mortality scores (Table 1). There was no difference in the proportion of hydroxyurea treatment or exchange transfusion program between patients with AO and the other patients. In addition, the proportion of patients that had been transfused prior to admission was similar between the two groups. Patients with AO had significantly higher white blood cell count on admission and were more likely to receive fluid resuscitation, intravenous antibiotics, enteral nutrition, and blood transfusion or exchange transfusion during the PICU stay (Table 2). There was no difference in the higher level of HbS during stay between patients with AO and other patients. Patients with AO required central venous access and arterial catheters in 25 et 27% respectively.

**Table 2:** Characteristics of pediatric intensive care unit stay of patients with sickle cell disease

	All patients (*N* = 579)	Patients with adverse outcome (*N* = 113)	Patients without adverse outcome (*N* = 466)	MD	p-value
Biological datas					
Maximum CRP (mg/L)	117 [54–189] (< 10–467)	126 [68–173] (< 10–409)	110 [50–193] (< 10–467)	100	NS
Minimal hemoglobin (g/dl)	8.0 [6.8-9.0] (2.3–12.9)	7.2 [6.1–8.3] (3.3–12.3)	8.2 [6.9–9.2] (2.3–12.9)	23	< 0.01
Maximum delta of hemoglobin during stay	0 [0.0-1.2] (-0.7-6.7)	0.1 [0.0-1.6] (0.0-3.9)	0.0 [0.0-1.1] (-0.7-6.7)	23	< 0.01
Maximal hemoglobin S percentage during stay (%)	58 [41–72] (7–94)	59 [40–78] (7–93)	57 [43–70] (12–94)	263	NS
Maximal white blood cell count (x10^**3**^**/mm** ^**3**^)	17.1 [13.0-23.3] (1.4–52.7)	21.0 [15.0-27.4] (2.8–52.7)	16.4 [12.8–22.1] (1.4–47.5)	19	< 0.01
Hyponatremia < 135 mmol/l, n (%)	155 (29%)	39 (36%)	119 (28%)	39	NS
Acute renal failure, n (%)	16 (3%)	8 (7%)	8 (2%)	29	*†*
Treatments					
Ventilatory support					
NIV, n (%)	291 (50%)	89 (79%)	202 (43%)	0	< 0.01
Duration (days)	2 [2–3] (0–9)	4 [4–5] (0–9)	2 [2–3] (0–3)	0	<0.01
MV, n (%)	47 (8%)	47 (41%)	0 (0%)	0	-
Duration (days)	3 [2–6] (0–27)	3 [2–6] (0–27)	-	0	
Nitric oxide, n (%)	20 (4%)	11 (10%)	8 (2%)	1	*†*
High-frequency ventilation, n (%)	6 (1%)	6 (5%)	0 (0%)	1	-
**Hemodynamic support, n (%)**					
Fluid resuscitation	13 (3%)	12 (12%)	1 (0%)	0	*†*
Vasoactive drugs	12 (2%)	12 (11%)	0 (0%)	0	-
**Anti-infective therapy**					
Intravenous antibiotics, n (%)	490 (85%)	111 (98%)	379 (82%)	1	< 0.01
Duration (days)	6 [3–8] (0–57)	8.5 [5–11] (0–57)	5.0 [3–7] (0–20)	2	< 0.01
**Hematologic support**					
Transfusion prior to admission, n (%)	211(38%)	43 (39%)	168 (38%)	39	NS
Number	1 [1–1] (1–3)	1 [1–1] (1–3)	1 [1–1] (1–3)	3	NS
Transfusion in PICU, n (%)	222 (38%)	59 (52%)	163 (35%)	1	< 0.01
Number	1 [1–1] (1–6)	1 [1–1] (1–6)	1 [1–1] (1–3)	17	< 0.01
Transfusion exchange in PICU, n (%)	155 (27%)	45 (40%)	110 (24%)	2	< 0.01
Number	1 [1–1] (1–3)	1 [1–1] (1–3)	1 [1–1] (1–2)	6	< 0.01
No transfusion at any time	129 (23%)	16 (14%)	113 (24%)	9	0.01
**Nutritional support, n (%)**					
Enteral nutrition	69 (12%)	31 (28%)	38 (8%)	9	< 0.01
Parenteral nutrition	6 (1%)	4 (4%)	2 (0%)	22	*†*
Renal replacement therapy, n (%)	3 (1%)	3 (3%)	0 (0%)	0	†
**Invasive device, n (%)**					
Central catheter	45 (8%)	28 (25%)	17 (4%)	1	< 0.01
Arterial catheter	68 (12%)	30 (27%)	38 (8%)	1	< 0.01
**Evolution**					
**Length of stay (days)**	3 [1–4] (0–57)	5 [4–7] (1–57)	2 [1–3] (0–16)	0	< 0.01
**Death during PICU stay, n (%)**	7 (1%)	7 (6%)	0 (0%)	0	-
Timing of death (days)	2 [1–7] (1–14)	2 [1–7] (1–14)	-		
**Death during 6 months follow up, n (%)**	7 (1%)	7 (6%)	0 (0%)	0	*-*

PICU : pediatric intensive care unit ; MD : Missing Data ; NIV : non-invasive ventilation ; MV : mechanical ventilation ; NS : non-significant. Adverse outcome (AO) was defined in case of NIV > 3 days (11), IMV (excluding the first 24 h after surgery), vasopressors, renal replacement therapy or in case of death in the PICU. *Acute anemia refers to an Hb level decrease ≥ 20% versus the baseline value and due to any reasons excepted splenic sequestration (Delayed Hemolytic Transfusion Reaction, Parvovirus infection…). † Irrelevant statistical testing due to reduced effectives Quantitative data are reported as median [interquartile range] (extreme values)

On multivariable analysis, baseline hemoglobin < 8 g/dl, history of bronchial obstruction and admission for ACS were associated with AO (Table 3).

**Table 3:** Univariate and multivariable analysis of factors associated with adverse outcome among patients with Sickle Cell Disease admitted in Pediatric Intensive Care Unit

	Univariate analysis		Multivariable analysis	
Variables	p-value	Crude OR [IC]95%	p-value	Adjusted OR [IC]95%
Baseline hemoglobin < 8 g/dl	< 0.01	1.06 [1.17–2.83]	**0.02**	**1.71 [1.07–2.73]**
G6PD deficiency	0.10	1.34 [0.90–2.84]	NS	
History of bronchial obstruction	0.01	1.81 [1.15–2.86]	**0.04**	**1.67 [1.05–2.66]**
Reason for admission (ACS versus other)	< 0.01	2.06 [1.31–3.23]	**0.01**	**1.80 [1.11–2.94]**

ACS : acute chest syndrome ; NS : non-significant

## Discussion

Our study suggested that children with baseline hemoglobin < 8 g/dl, history of bronchial obstruction and admission for ACS experienced more AO. Overall, patients with SCD admitted to the PICU for an acute complication required NIV in 50% of cases, MV in 8% of cases and the overall mortality was low (1%). Sepsis was uncommon but was the leading cause of mortality.

As in adults, the two main reasons for admission in PICU were ACS and severe VOPC [6]. Sepsis in our population was rare (4%) and confirms the decreasing trend of severe infections in children with SCD thanks to prophylactic penicillin and vaccination [17, 18]. In fact, in a recent multicenter retrospective cohort study that included patients with SCD younger than 22 years (young adults) presenting to emergency departments with fever found that bacteriemia was found only in 1.1% of patients (95% CI, 1.05-1.26%) [19].

The overall mortality rate was lower compared to the critically ill SCD adult patients [6, 20] and to the general PICU population [21]. This in accordance with two previous multicenter pediatric study that showed that the overall mortality of SCD patients admitted to PICU was low but was variable between centers, highly influenced by the volume of patients admitted [8, 22]. These patients mostly survive after PICU admission but nevertheless during their stay, they experienced many well-known risk factors of post-intensive care syndrome (PICS) like invasive or non-invasive ventilatory support, inadequate pain control and immobility [23]. This population requires a particular attention after PICU discharge, and the follow-up should be multidisciplinary. Moreover, apart from the consequence on the child’s health, this population is at high risk of being readmitted to the PICU. In fact, 29% of the patients in our cohort had several stays which is in line with a study on SCD adult patients that found that 30% of the survivors were re-admitted to the ICU within the following year [6].

Patient with baseline hemoglobin > 8 g/dL experienced less AO which is important to take into consideration knowing the effect of hydroxyurea treatment on hemoglobin levels [24, 25]. However, in our cohort, only 51% of the population was treated with hydroxyurea with no difference in frequency between groups, which suggest that other factors are involved, like more intense hemolysis and disease activity. Nevertheless, early initiation of hydroxyurea using individualized, pharmacokinetics-guided is more and more widely performed [26] and this might improve outcomes, including cerebral vasculopathy [27]. In addition, a recent study has shown that higher dose of hydroxyurea was more efficient on fetal hemoglobin induction and on the onset of vaso-occlusive events without significant toxicity in young children [28].

Furthermore, patients with ACS experienced more AO, requiring more life-supporting treatments, which is in line with previously published studies that found that ACS was the main cause of death in SCD patients [29] and that children with ACS required MV or NIV in 6% of the cases [7]. In fact, phenotypes of rapidly progressive ACS have been described and associated with a high rate of multiorgan failure but is more frequent in adults [30]. Although this is a well-known complication of patients with SCD with high morbidity, there is still a lack of high-quality studies to improve the prognostic of patients [31]. In fact, the existing recommendation of care still relies on low quality evidence [32] and further randomized controlled trials are urgently needed. In addition, we also found that a history of bronchial obstruction was associated with adverse outcome. This is probably related to the association with ACS since the association between bronchial obstruction and ACS severity, ACS recurrence and earlier death have also been previously reported [33]. Even if the associated factors and clinics between ACS and asthma can overlap, bronchial obstruction is a distinct entity, and must be closely monitored and controlled in patients with SCD given these results.

Finally, it is interesting to note that patients with AO did not differ from the other less severe patients regarding the frequency of transfusion prior to PICU transfer. In fact, with the growing number of patients developing delayed hemolytic transfusion reactions [34, 35], this result could be taken into account when evaluating the benefice-risk balance of red blood cell transfusion and other risk factors should be taken into consideration to intensify the patient’s treatment. In our study, HbS level was not significantly different between the two groups. Despite some limitation discussed below, this is important for clinicians to acknowledge as some very severe patients might present with low HbS. This is consistent with the fact that HbS is not the only pathological issue in these patients who experience a systemic disease involving endothelium and inflammatory pathways [36, 37].

The main limitation of our study is its retrospective and monocentric design. In fact, the study has been performed in a tertiary hospital that is a reference center for pediatric patients with SCD and this might have introduced a bias in the epidemiology, especially regarding mortality. However, the large sample size with very few missing data and detailed description of patient’s characteristics provides an accurate description of clinical and biological characteristics, PICU treatments, and outcomes, that could be useful for both clinician in their daily practice and for future therapeutic studies. Finally, we chose a composite criterion to identify patients with AO that included NIV for more than 3 days according to previous publications that found that most patients with ACS were ventilated for less than 3 days [16]. The external validity of the study might impacted as some centers use NIV early in the course of disease independently from the patients severity [12].

## Conclusion

Baseline hemoglobin < 8 g/dL, history of bronchial obstruction and admission for ACS were strong risk factors for severe evolution in SCD children admitted to PICU during the last decade. These factors could be taken into consideration when choosing the adequate therapeutic options. In addition, given the low mortality and the fact that these patients experience during their stay many well-known risk factors of PICS, systematic screening regarding this aspect should be performed during follow-up.

### Electronic supplementary material

Below is the link to the electronic supplementary material.

Supplementary Material 1

## Data Availability

The datasets used and/or analyzed during the current study are available from the corresponding author on reasonable request.

## Declarations

(n° 2022 − 635).

## Abbreviations

ACS: Acute Chest Syndrome
AO: Adverse Outcome
HSCT: Hematopoietic Stem Cell Transplantation
LOS: Length Of Stay
MV: Invasive Mechanical Ventilation
NIV: Non-Invasive Ventilation
PICS: Post-Intensive Care Syndrome
PICU: Pediatric Intensive Care Unit
SCD: Sickle Cell Disease
VOPC: Vaso-Occlusive Painful Crisis

## References

[1] Kavanagh PL, Fasipe TA, Wun T (2022). Sickle cell disease: a review. JAMA.

[2] GBD 2021 Sickle Cell Disease Collaborators (2023). Global, regional, and national prevalence and mortality burden of sickle cell disease, 2000–2021: a systematic analysis from the global burden of Disease Study 2021. Lancet Haematol.

[3] Telfer P, Coen P, Chakravorty S, Wilkey O, Evans J, Newell H (2007). Clinical outcomes in children with sickle cell disease living in England: a neonatal cohort in East London. Haematologica.

[4] Quinn CT, Rogers ZR, McCavit TL, Buchanan GR (2010). Improved survival of children and adolescents with sickle cell disease. Blood.

[5] Cecchini J, Lionnet F, Djibré M, Parrot A, Stojanovic KS, Girot R (2014). Outcomes of adult patients with sickle cell disease admitted to the ICU: a case series*. Crit Care Med.

[6] Agbakou M, Mekontso-Dessap A, Pere M, Voiriot G, Picard M, Bourenne J (2021). Nationwide retrospective study of critically ill adults with sickle cell disease in France. Sci Rep.

[7] Takahashi T, Okubo Y, Pereda MA, Handa A, Miller S (2018). Factors Associated with mechanical ventilation use in children with sickle cell disease and acute chest syndrome. Pediatr Crit Care Med J Soc Crit Care Med World Fed Pediatr Intensive Crit Care Soc.

[8] Ettinger NA, Guffey D, Anum SJ, Fasipe T, Katkin J, Bhar S (2023). Multi-center retrospective study of children with sickle cell disease admitted to pediatric intensive care units in the United States. Sci Rep.

[9] Cieza-Asenjo R, García-Morín M, Escobar-Fernández L, Cela-de Julián E, Slöcker-Barrio M, Herrera-Castillo L (2022). Sickle cell disease in pediatric intensive care. Pediatr.

[10] Bartram JL, Thein SL, Gardner K, Egberongbe Y, D’Silva P, Height SE (2010). Outcome of children with sickle cell disease admitted to intensive care - a single institution experience. Br J Haematol.

[11] Yousef AA, Shash HA, Almajid AN, Binammar AA, Almusabeh HA, Alshaqaq HM (2022). Acute chest syndrome in pediatric sickle cell disease: a 19-year tertiary center experience. Ann Thorac Med.

[12] Heilbronner C, Merckx A, Brousse V, Allali S, Hubert P, de Montalembert M (2018). Early noninvasive ventilation and nonroutine transfusion for acute chest syndrome in Sickle Cell Disease in children: a descriptive study. Pediatr Crit Care Med J Soc Crit Care Med World Fed Pediatr Intensive Crit Care Soc.

[13] Sommet J, Alberti C, Couque N, Verlhac S, Haouari Z, Mohamed D (2016). Clinical and haematological risk factors for cerebral macrovasculopathy in a sickle cell disease newborn cohort: a prospective study. Br J Haematol.

[14] Beydon N, Davis SD, Lombardi E, Allen JL, Arets HGM, Aurora P (2007). An official American Thoracic Society/European Respiratory Society statement: pulmonary function testing in preschool children. Am J Respir Crit Care Med.

[15] Pellegrino R, Viegi G, Brusasco V, Crapo RO, Burgos F, Casaburi R (2005). Interpretative strategies for lung function tests. Eur Respir J.

[16] Idier C, Leger P-L, Levy M, Rambaud J, Dauger S. Non-invasive ventilation in paediatric acute chest syndrome (NIVIPACS): a preliminary two-centre prospective observational study. Minerva Anestesiol. 2020. 10.23736/S0375-9393.20.14972-1 33300327

[17] Yee ME, Lai KW, Bakshi N, Grossman JK, Jaggi P, Mallis A (2022). Bloodstream infections in Children with Sickle Cell Disease: 2010–2019. Pediatrics.

[18] Ochocinski D, Dalal M, Black LV, Carr S, Lew J, Sullivan K (2020). Life-threatening infectious complications in Sickle Cell Disease: a concise narrative review. Front Pediatr.

[19] Rineer S, Walsh PS, Smart LR, Harun N, Schnadower D, Lipshaw MJ (2023). Risk of Bacteremia in Febrile Children and Young adults with Sickle Cell Disease in a Multicenter Emergency Department Cohort. JAMA Netw Open.

[20] Gardner K, Bell C, Bartram JL, Allman M, Awogbade M, Rees DC (2010). Outcome of adults with sickle cell disease admitted to critical care - experience of a single institution in the UK. Br J Haematol.

[21] Leteurtre S, Duhamel A, Salleron J, Grandbastien B, Lacroix J, Leclerc F (2013). PELOD-2: an update of the PEdiatric logistic organ dysfunction score. Crit Care Med.

[22] Heilbronner C, Grimaud M, Oualha M, Sommet J, Rambaud J, Brousse V (2021). Therapeutic approach to pediatric patients with acute chest syndrome: national multicenter survey of non invasive ventilation (NIV) and transfusion. Arch Pediatr Organe off Soc Francaise Pediatr.

[23] Woodruff AG, Choong K (2021). Long-term outcomes and the Post-intensive Care Syndrome in critically Ill children: a north American perspective. Child Basel Switz.

[24] Rodriguez A, Duez P, Dedeken L, Cotton F, Ferster A (2018). Hydroxyurea (hydroxycarbamide) genotoxicity in pediatric patients with sickle cell disease. Pediatr Blood Cancer.

[25] Rankine-Mullings AE, Nevitt SJ (2022). Hydroxyurea (hydroxycarbamide) for sickle cell disease. Cochrane Database Syst Rev.

[26] Quinn CT, Niss O, Dong M, Pfeiffer A, Korpik J, Reynaud M (2021). Early initiation of hydroxyurea (hydroxycarbamide) using individualised, pharmacokinetics-guided dosing can produce sustained and nearly pancellular expression of fetal haemoglobin in children with sickle cell anaemia. Br J Haematol.

[27] Karkoska K, Pfeiffer A, Beebe DW, Quinn CT, Niss O, McGann PT (2022). Early hydroxyurea use is neuroprotective in children with sickle cell anemia. Am J Hematol.

[28] Wang W, Brown C, McNaull M, Rogers ZR, Barton M, Dua M et al. Intensive hydroxyurea dosing in very young children with sickle cell anemia. Blood Adv. 2023;bloodadvances.2022009613. 10.1182/bloodadvances.2022009613 PMC10685164 37695741

[29] Pinto VM, Gianesin B, Piel FB, Longo F, Rigano P, Quota A et al. Morbidity and mortality of sickle cell disease patients is unaffected by splenectomy: evidence from 3 decades follow-up in a high-income setting. Haematologica. 2022. 10.3324/haematol.2022.280815 PMC10071109 35924578

[30] Chaturvedi S, Ghafuri DL, Glassberg J, Kassim AA, Rodeghier M, DeBaun MR (2016). Rapidly progressive acute chest syndrome in individuals with sickle cell anemia: a distinct acute chest syndrome phenotype. Am J Hematol.

[31] Niazi MRK, Chukkalore D, Jahangir A, Sahra S, Macdougall K, Rehan M (2022). Management of acute chest syndrome in patients with sickle cell disease: a systematic review of randomized clinical trials. Expert Rev Hematol.

[32] Bhasin N, Sarode R. Acute chest syndrome in Sickle Cell Disease. Transfus Med Rev. 2023;150755. 10.1016/j.tmrv.2023.150755 37741793

[33] DeBaun MR, Strunk RC (2016). The intersection between asthma and acute chest syndrome in children with sickle-cell anaemia. Lancet Lond Engl.

[34] Rossi M, Pirenne F, Le Roux E, Smaïne D, Belloy M, Eyssette-Guerreau S (2023). Delayed haemolytic transfusion reaction in paediatric patients with sickle cell disease: a retrospective study in a French national reference centre. Br J Haematol.

[35] Fasano RM, Miller MJ, Chonat S, Stowell SR (2019). Clinical presentation of delayed hemolytic transfusion reactions and hyperhemolysis in sickle cell disease. Transfus Clin Biol J Soc Francaise Transfus Sang.

[36] Hoppe CC (2014). Inflammatory mediators of endothelial injury in sickle cell disease. Hematol Oncol Clin North Am.

[37] Zhang D, Xu C, Manwani D, Frenette PS (2016). Neutrophils, platelets, and inflammatory pathways at the nexus of sickle cell disease pathophysiology. Blood.

