# Supplementary material for: Factors associated with adverse outcome among children with sickle cell disease admitted to the pediatric intensive care unit: an observational cohort
Source: Ann Intensive Care. 2024 Apr 10;14:55. doi: 10.1186/s13613-024-01283-5 (PMC11003929; doi:10.1186/s13613-024-01283-5)
Supplement: Supplementary file 1 — Supplementary Material 1 [file 13613_2024_1283_MOESM1_ESM.docx]

Michael Levy, MD, PhD

Service de Médecine Intensive et

Réanimation Pédiatriques

Hôpital Robert-Debré

48, Bd Sérurier

75019 Paris, France

Tel.: +33 140 032 287

Fax: +33 140 034 760

E-mail: [michael.levy@aphp.fr](mailto:michael.levy@aphp.fr)

Paris, March 13, 2024

**Factors associated with adverse outcome among children with sickle cell disease admitted to the Pediatric Intensive Care Unit: an observational cohort (AOIC-D-23-00746R1)**

Dear Editor-in-Chief,

We thank you again for considering our manuscript for publication in *Annals of Intensive Care.* Following the recommendations made on the second review of our manuscript, we are resubmitting the enclosed manuscript for your consideration for publication in *Annals of Intensive Care* as a *Research article.*

Our thanks also go to the Reviewers and Editor whose reviewing process considerably improved the quality of the paper. We have addressed all the points raised and provide below a point-by point response explaining how we dealt with the comments of the Reviewers and Editor.

The modifications appear in blue in the revised version of the manuscript.

We hope that the revised version meets the concerns of the Editor and Reviewers.

Best regards

Michaël LEVY

**Comments:**

**Reviewer #1:**

I would like to thank the authors for their answers to the previous queries I made. I understand that the study beeing retrospective, some data might be missing. 
I found this study very interesting
A few minor changes might be suggested
We thank again the Reviewer #1 for his great review of our work and all his constructive comments. We provide below a point-by-point reply.

L 179: refrain form adverbs in the results section ("finally"), especially since it is not final (an other result comes after) 
We deleted this adverb accordingly line 180.

 L 185: demonstrated might be modified (suggested/ implies/...), authors should be a little more cautious with the results because of this retrospective design

We replaced “demonstrated” by “suggested” line 185.

L200 et sqq: you did not describe actual PICS in your study but you would like it to be screened and adressed, the  formulation is a little redundand /heavy, too many "thus" for instance, it might be rephrased
 "these patients mostly survive after admission to the PICU for acute crisis and are particularly at risk of developing chronic morbidities like post-intensive care syndrome (PICS). In fact, these patients experience during their stay many well-known risk factors of PICS larticularly at risk of developing chronic morbidities like post-intensive care syndrome (PICS).In fact, these patients experience during their stay many well-known risk factors of PICS like invasive or  non-invasive ventilatory support, inadequate pain control and immobility [23]
Something like "These patients mostly survive after admission to the PICU but nevertheless during their stay they experienced many well-known risk factors of post-intensive care syndrome (PICS) like invasive or non-invasive ventilatory support, inadequate pain control and immobility [23].
Thank you for this suggestion. We changed the formulation as suggested line 200 and deleted ‘thus” line 207.

About Hydroxyurea, you could add somewhere (methods? discussion?) a sentence about compliance to the treatment (something like "the study beeing retrospective, actual patient adherence to treatment wasn't known ")

We added this sentence in the method section, line 110 “Given the retrospective design of the study, compliance with treatments could not be assessed”.


L 237 et sqq: I recommand beeing cautious about the formulation (retrospective, missing data for most patients) but also authors should insist on the clinical importance of this finding 
For instance " In our study HbS level was not significantly different between the two groups. Although retrospective and with some missing data, this is important for clinicians to acknowledge as some very severe patients might present with low HbS. This is consistent with the fact that HbS is not the only pathological issue in these patients who experience a systemic disease involving endothelium and inflammatory pathways.

Thank you for this suggestion, we replaced «  Furthermore, it is interesting to see that patients with AO did not have significantly higher levels of HbS levels than the other patients, confirming that SCD is also a systemic disease involving endothelium and inflammatory pathways” by « In our study, HbS level was not significantly different between the two groups. Despite some limitation discussed below, this is important for clinicians to acknowledge as some very severe patients might present with low HbS. This is consistent with the fact that HbS is not the only pathological issue in these patients who experience a systemic disease involving endothelium and inflammatory pathways [36,37].” line 235


**Reviewer #2:**

I thank the authors for having taken in consideration the comments and having modified their manuscript accordingly when possible.

Thank you again for your review of our manuscript and your time.
